# Effects of Yiqi Tongyang on HCN4 Protein Phosphorylation in Damaged Rabbit Sinoatrial Node Cells

**DOI:** 10.1155/2016/4379139

**Published:** 2016-03-16

**Authors:** Jinfeng Liu, Ruxiu Liu, Jie Peng, Yanli Wang

**Affiliations:** Department of Cardiovascular, Guang'anmen Hospital, China Academy of Chinese Medical Sciences, Beijing 100053, China

## Abstract

The hyperpolarization-activated cyclic nucleotide-gated cation channel (*I*_*f*_) is closely associated with sinoatrial node pacing function. The present study aimed to investigate the molecular mechanisms involved in pacing function improvements of damaged sinoatrial node cells and the consequent treatment effects on sick sinus syndrome (SSS) after the use of Yiqi Tongyang. HCN4 channel protein expression and phosphorylation were measured by immunoblotting and fluorescent quantitation. After ischemia-reperfusion injury (model group), the HCN4 protein and the optical density (OD) of the phosphorylated HCN4 protein as well as intracellular PKA activity in the sinoatrial node cells decreased significantly. However, the OD values and PKA activity increased to different degrees after treatment with serum containing different doses of Yiqi Tongyang; in contrast, no significant improvement was seen in the control group compared to the model group. These findings demonstrated that the use of the traditional Chinese medicine Yiqi Tongyang could increase HCN4 protein expression and phosphorylation as well as PKA activity within sinoatrial node cells damaged by ischemia-reperfusion. The HCN4 protein is involved in the *I*_*f*_-related ion channel. Here, we speculated that these effects could be associated with upregulation of HCN4 protein phosphorylation, which consequently improved cell automaticity, increased heart rate, and had treatment effects on SSS.

## 1. Introduction

Sick sinus syndrome (SSS) is a group of arrhythmias induced by pacing and/or conduction dysfunction caused by lesions within the sinus sinoatrial node and surrounding tissues. Bradyarrhythmia is the major clinical presentation of SSS, and there is currently no effective drug therapy for it in western medicine. Although the use of a cardiac pacemaker in patients with serious disease could partially alleviate symptoms and improve quality of life, patient mortality rates have not decreased significantly [1]. In addition, the medical costs of this method are relatively high and the existence of multiple contradictions and complications has prevented its wide application. Cardiac biological pacing has emerged as a hotspot in recent years; however, most studies focusing on cardiac biological pacing were performed in* in vitro* or animal experiments [2]. In contrast, traditional Chinese medicine (TCM) is a reliable and cost-effective method for the treatment of SSS.

Studies have shown that the pacing function of the sinoatrial node is closely associated with the hyperpolarization-activated cyclic nucleotide-gated cation channel (*I*_*f*_). The pacemaker current channel (*I*_*f*_ channel) plays a crucial role in the maintenance of sinoatrial node cell (SNC) automaticity. Studies have shown that the development of SSS is directly associated with structural damage as well as decreased SNC activity and automaticity, of which the molecular basis is the reduced automaticity caused by the structural abnormality of the *I*_*f*_ channel [3]. The activity of HCN4, the major component of the *I*_*f*_ channel, is dependent on its phosphorylation level, which is regulated by the protein kinase A (PKA) signaling pathway [4]. In recent years, Yiqi Tongyang, developed by Zhiming Liu, a TCM master, has been used to treat SSS, and clinical research results to date have demonstrated its efficacy and safety. Here, we further investigated HCN4 channel protein expression and phosphorylation to explore the mechanisms involved in the treatment effects of Yiqi Tongyang for SSS.

## 2. Materials and Methods

### 2.1. Animals

Healthy New Zealand rabbits (body weight: 250–300 g) of both sexes were obtained within 24 h after birth from Beijing Longan experimental animal farm (number 11401400000095). SNC were isolated and identified by morphological and electrophysiological examinations. The rabbits were divided into four groups: normal (control), normal saline, serum control (serum without Yiqi Tongyang), and Yiqi Tongyang (serum containing Yiqi Tongyang). Ischemia-reperfusion was used for all rabbits except those in the control group to induce SNC damage.

### 2.2. Drugs and Reagents

Serum containing Yiqi Tongyang (drugs including* Aconitum napellus*, Radix Ginseng Rubra,* Panax pseudoginseng*, and Radix Glycyrrhizae Preparata) and serum without Yiqi Tongyang were prepared in-house. Dulbecco's modified Eagle medium supplemented with F12 (DMEM/F12, 1 : 1) culture medium was obtained from Thermo (USA), low-glucose DMEM culture medium was purchased from Hyclone (USA), and fetal bovine serum (FBS) was obtained from Gibco (USA). Trypsin, collagenase II, and Triton X-100 were purchased from Sigma (USA), and PIPA lysis buffer and PMSF were from Beijing Dingguo Changsheng Biotechnology Co., Ltd. (China). Polyvinylidene fluoride (PVDF) membranes, nitrocellulose membranes, electrophoresis buffer, transfer buffer, 20x Tris-buffered saline (TBS), and 1x TBS containing 0.05% Tween-20 (TBST) were also used. Mounting buffer was prepared by dissolving 5% skim milk powder in the TBST. ECL chemiluminescence kit was also used. PKA activity was quantified using GENMED kits.

### 2.3. Equipment

The used instruments included ultrapure water equipment (Millipore, USA), gel imaging system (Fujifilm LAS-4000, Japan), gel electrophoresis system (Amersham Pharmacia Biotech EPS301, USA), transfer film system (Bio-Rad Trans-Blot® SD Semi-Dry Transfer Cell, USA), low speed centrifuge (Universal 32R, Germany), electronic balance (Mettler Toledo, Switzerland), and optical microscope (Olympus CK40, Japan).

### 2.4. Preparation of Serum with or without Yiqi Tongyang

Serum was prepared as we reported previously [5]. Forty New Zealand white rabbits (males = 20, females = 20) were divided into four groups using a random number table according to body weight with 10 rabbits in each group. Sera with different doses of Yiqi Tongyang were then prepared. Briefly, crude Yiqi Tongyang drugs were dissolved in distilled water to obtain different doses (high, moderate, and low of 54.96, 27.48, and 13.74 g/kg body weight, resp.) in a suspension equal to 12-, 6-, and 3-fold the clinical dose for a 70 kg person, respectively. Gavage administration of Yiqi Tongyang suspension was performed twice/day for 7 continuous days in each rabbit. To create serum without Yiqi Tongyang, the same volume of drinking water was provided for the rabbits. Drinking water but no food was provided for the rabbits within 12 h before the last administration; blood was obtained from the central ear artery at 2 h after the last administration, and the serum was obtained after natural separation. The serum was inactivated by placement in 56°C water for 30 min, followed by filtration through a 0.2 *μ*m mesh. The serum was stored at −20°C until use.

### 2.5. SNC Separation and Culture

SNC were separated from newborn rabbits and cultured as we reported previously [5]. In brief, four newborn New Zealand rabbits (within 24 h after birth) were anesthetized by ether inhalation, and 75% ethanol was used to disinfect the chest. The chest was cut open along the thoracic midline to expose the heart. The major blood vessels were resected from the cardiac base to obtain the heart but preserved whenever possible. The heart was rinsed with PBS on ice until no blood remained. The sinoatrial node region (conjunction between the superior vena cava and right atrium) was separated under an anatomical lens. The tissues were rinsed with PBS and then cut into small pieces (about 1 mm^3^). The pieces were placed in 10 mL of 0.1% trypsin, incubated in a 37°C shaking water bath for 5 min, pipetted vigorously for 1 min, and allowed to precipitate and the supernatant was discarded. Collagenase II (0.025%) was added and the mixture was incubated in a 37°C shaking water bath for 10 min and precipitated and the supernatant was collected in a 50 mL centrifugation tube. DMEM (20 mL) containing 15% FBS was added to the tube, while the remaining tissues were digested repeatedly until single cells were obtained. The cell suspension was also collected in the centrifugation tube. After centrifugation at 1,500 r/min for 10 min, the supernatant was discarded, and 6 mL of the culture medium containing serum was used to resuspend the cells. The cell suspension was evenly divided into four portions and then seeded into four 10 cm culture dishes containing 10 mL of culture medium. The culture dishes were incubated at 37°C under 5% CO_2_ for 1 h. The differential velocity adherent technique and 5-BrdU were used to obtain purified SNC. Microscopic observation revealed large amount of fibrocytes and interstitial cells adhering to the dish; the unattached cells were collected and cultured in another 4 dishes for further culture. The medium was changed 24 h later and then every 48 h thereafter.

### 2.6. Model Induction

The model was induced according to the method established in our previous studies [5], the method used by Bielawska et al. [6] to induce myocardial apoptosis, and the method used by Koyama et al. [7] to induce ischemia-reperfusion of primary SNC with modifications. Oxygen and glucose deprivation was used to mimic ischemia, while resuming oxygen and glucose was used to mimic reperfusion. The detailed methods were as follows: (1) SNC were digested, collected, and seeded in 24-well culture dishes with sterilized coverslip for culture (100 cells were randomly selected and counted under an inverted microscope 5 d later, and cells in which spindle-shaped cells accounted for more than 60% were selected for the following experiments); (2) SNC were preincubated with DMEM in an aerobic environment for 30 min before ischemia-reperfusion damage was induced (the time for ischemia was 60 min, while that for reperfusion was 3 h); and (3) the culture medium was changed to N_2_-saturated serum-deprived medium, and the cells were placed in a custom air-proof box, into which 30 volumes (about 10 L) of mixed air containing 95% N_2_ and 5% CO_2_ were ventilated to completely remove the residual oxygen from the box, which was then placed into the incubator for 60 min to mimic cellular ischemia; and (4) the cells were removed from the box, the medium was replaced by mimic reperfusion medium to replenish the oxygen and glucose, and the cells were incubated in a 5% CO_2_ incubator for 3 h.

### 2.7. Measuring HCN4 Protein Expression and Phosphorylation

HCN4 protein phosphorylation was measured as reported by Lin et al. [8].

#### 2.7.1. Measuring HCN4 Expression with Western Blotting

(1) The SNC in each group were collected in a centrifugation tube, and cell lysis buffer (500 *μ*L/2 × 10^7^ cells) was added to extract the total protein, which was preserved at −20°C; and (2) protein concentrations were quantified by the bichionic acid (BCA) method. Total protein (15 g) was obtained for 10% sodium dodecyl sulfate- (SDS-) PAGE electrophoresis. The protein on the gel was transferred to a PVDF membrane, mounted with 5% skim milk powder at room temperature for 1 h, and incubated with primary antibody at 4°C overnight. The membrane was washed with PBST three times at room temperature and then incubated with secondary antibody at 37°C for 1 h. The membrane was washed with PBST three times at room temperature, followed by one wash with PBS, and then exposed by ELC and developed; and (3) the antibody on the same PVDF membrane was removed, incubated with *β*-actin antibody, developed, and used as an internal reference. The intensity was calculated according to the following formula: intensity = area × gray scale. Protein intensity was compared to that of *β*-actin of the same sample.

#### 2.7.2. Measuring HCN4 Protein Phosphorylation with Immunoprecipitation and Western Blotting

(1) Cell lysis kits were used to extract the total protein, membrane protein extraction kits were used to break the membrane, and the Bradford or BCA method was used for the quantification of the protein concentration; and (2) antibodies against the proteins with tyrosine phosphorylation (4G10) were added and incubated at 4°C for 1 h, to which protein A/G agarose was added, and the mixture was shaken gently and incubated overnight, followed by three washes with precooled PBS, and immunoprecipitation was then performed after resuspension with 2x sample buffer; and (3) for western blotting 5–20 *μ*g of the protein sample was added to 4–12% SDS-PAGE for electrophoresis, followed by transmembrane incubation with anti-HCN4 primary antibody, washing, incubation with horseradish peroxidase binding secondary antibody, and developing.

#### 2.7.3. Measuring PKA Activity

Fluorescence quantification of the PKA activity was performed using quantification kits (Shanghai Jiemei).

### 2.8. Statistical Analyses

SPSS 13.0 software was used for the statistical analyses. Quantitative data are presented as means and standard divisions. Comparisons among different groups were performed using analysis of variance, while a post hoc least significant differences test was performed for pairwise analyses. Values of *P* < 0.05 were considered statistically significant.

## 3. Results

### 3.1. Typical Rabbit SNC

**Figure 1:**
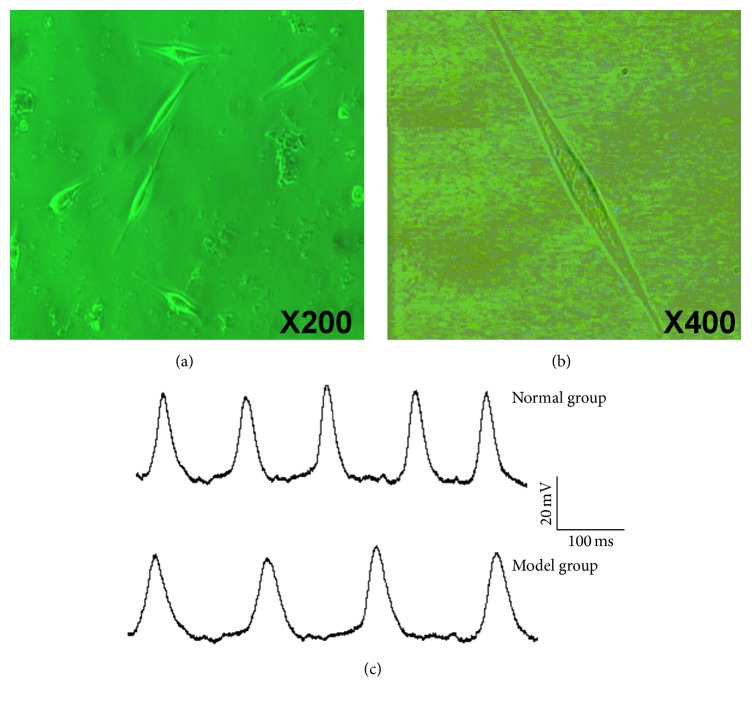
Sinoatrial node cells of the newborn rabbits. (a) Sinoatrial node cells at ×200. (b) Sinoatrial node cells at ×400. (c) Electrophysiological examination showing the spontaneous action potential of the newborn rabbit sinoatrial node cells.

The differential velocity adherent technique and 5-BrdU were used to obtain purified rabbit SNC. When observed under an inverted microscope, the cultured SNC showed spindle, triangular, and polygon shapes, and the percentage of the former was the highest (Figures 1(a) and 1(b)). The whole-cell mode of the current clamp technique, which was used to examine the action potential of the spindle-shaped cells, showed spontaneous depolarization in the diastolic phase with a mean maximum diastolic potential of −50.9 ± 4.8 mV and action potential amplitude of 62.9 ± 5.0 mV (Figure 1(c)). These cellular features confirmed that the cells were SNC.

### 3.2. HCN4 Protein Expression and Phosphorylation

**Figure 2:**
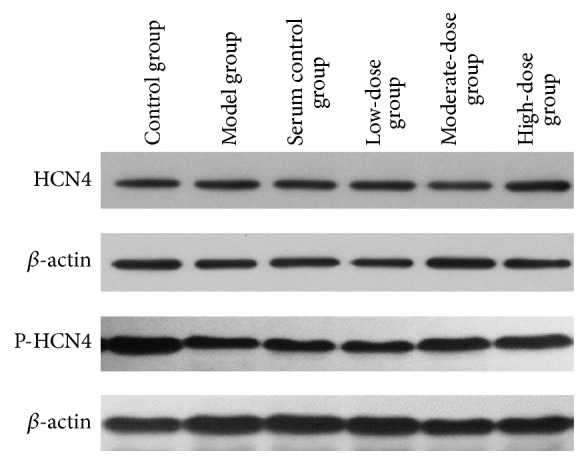
HCN4 protein expression and phosphorylation in rabbit sinoatrial node cells in each group.

The optical density (OD) value of the HCN4 protein expression and phosphorylation in the control group was 100, which was significantly higher than in the model group (42.47 ± 4.96) (*P* < 0.05), suggesting that the HCN4 protein expression and phosphorylation of the SNC decreased significantly after the model induction; thus, the ischemia-reperfusion model in the present study was applicable for the experiments. Compared with the model group, the cells treated with different doses of Yiqi Tongyang had higher OD values. Specifically, the cells treated with serum with moderate (114.55 ± 7.55) and high (129.14 ± 12.95) doses of Yiqi Tongyang had significantly higher OD values than those in the model group (*P* < 0.01), while the OD values of the cells treated with serum without Yiqi Tongyang (37.88 ± 6.24) or with a low dose of Yiqi Tongyang (45.46 ± 10.57) were not significantly different from those of the model group (*P* > 0.05). These findings suggest that Yiqi Tongyang could protect the damaged sinoatrial node tissues and increase the expression of phosphorylated HCN4 protein in the SNC. Pairwise comparisons showed that the OD values of the high- and moderate-dose groups were significantly higher than those of the low-dose and serum control groups (*P* < 0.05) in a dose-dependent manner. However, no significant difference was found between the low-dose and serum control groups (*P* > 0.05) (Table 1, Figure 2).

### 3.3. PKA Activities

The PKA activity in the control group was 0.18 ± 0.04 *μ*mol NADH/min, which was significantly higher than that in the model group (0.03 ± 0.00 *μ*mol NADH/min, *P* < 0.05), suggesting that the PKA activities in the SNC decreased significantly after the model induction. Compared with the model group, the PKA activities in the high- (0.11 ± 0.01 *μ*mol NADH/min), moderate- (0.06 ± 0.00 *μ*mol NADH/min), and low- (0.05 ± 0.00 *μ*mol NADH/min) dose groups were significantly higher (*P* < 0.01). In contrast, no significant difference was found between the serum control group (0.03 ± 0.00 *μ*mol NADH/min) and the model group (*P* > 0.05), suggesting that Yiqi Tongyang could significantly increase the PKA activities. In addition, pairwise comparisons showed that the effects were significantly better in the high-dose group than in the moderate- and low-dose groups as well as the serum group (*P* < 0.05) in a dose-dependent manner (Table 2).

## 4. Discussion

Studies have suggested that the pacemaker current (*I*_*f*_) could help increase the heart rate in acute stress responses of the sympathetic nerve. The *I*_*f*_ pathway is coded by the HCN gene family, which belongs to the hyperpolarization-activated cation channel. *I*_*f*_ is the major ion current in phase IV spontaneous depolarization of the SNC and the basis of cellular automaticity. *I*_*f*_ could be regulated by cyclic adenosine monophosphate (cAMP), while hormones and neurotransmitters could increase the cellular cAMP level, thus shifting the midpoint of the activation potential to a more depolarized potential as well as the dynamic characteristics of the channels [9]; therefore, sympathetic nerve stimulation and vagus nerve tension could increase or decrease the heart rate via cAMP-mediated *I*_*f*_. In addition, *I*_*f*_ has also been acknowledged as an important mechanism for coping with adrenaline-induced sinus tachycardia induced.

Studies have shown that the excitatory regulative effects on *I*_*f*_ induced by the sympathetic nerves are exerted through the direct binding of cAMP to the cyclic nucleotide binding domain at the -COOH end of the HCN4 channel, the processes of which are not dependent on HCN4 phosphorylation. However, several recent studies have shown that PKA activation is necessary for the sympathetic nerve regulation of *I*_*f*_. PKA could directly phosphorylate HCN4 and, thus, regulate ion current changes [10]. In addition, *I*_*f*_ has also been acknowledged as the end effect of cAMP signaling in SNC [11]. At least 13 residues were identified in the intracellular N and C termini of HCN4 that can be phosphorylated by PKA* in vitro*. Functional analysis of mutant channels showed that a PKA regulatory site in the distal C terminus of HCN4 is required for PKA modulation of *I*_*f*_. It suggests that *β* adrenergic regulation of *I*_*f*_ in the sinoatrial node may occur via multiple cAMP-dependent mechanisms. Regulation of cardiac and neuronal HCN channels by PKA may depend on the cellular context and/or the HCN channel isoform expression [12]. Therefore, abnormal HCN4 protein phosphorylation in SNC is closely associated with sinoatrial node dysfunction and could be one of the major molecular bases of SSS.

The Yiqi Tongyang recipe is an empirical formula invented by Zhiming Liu, a TCM master. This recipe has been used in clinical practice for more than 20 years, and its distinct efficacies have made it a popular recipe with patients. Liu's theory highlighted the renal origin of cardiovascular and cerebrovascular diseases. He suggested that the human pulse originated from the kidneys and merged in the heart. Since patients with SSS generally have an abnormally slow pulse, heart palpitations, dizziness, chest distress, and shortness of breath, a heart-kidney Yang deficiency has been acknowledged as one of the critical pathogeneses for which both the heart and kidneys should be treated to relieve both primary and secondary symptoms. The Yiqi Tongyang recipe mainly contains ginseng,* Aconitum napellus*, Radix Ginseng Rubra, and Radix Glycyrrhizae Preparata. The ginseng and* Aconitum napellus* in the recipe could benefit the qi, warm the Yang, and regulate the pulse, while the Radix Glycyrrhizae Preparata could benefit the qi, warm the Yang, regulate the pulse, and stop the palpitations. The concomitant use of Radix Glycyrrhizae Preparata, ginseng, and* Aconitum napellus* could result in Yang-Oriented Xin-Gan, which further improves the cardiac Yang. Radix Ginseng Rubra in the recipe could activate the blood, promote the meridian, soothe the nerves, and quiet the heart; thus, it could dilate the coronary artery, increase the blood flow in the coronary artery, decrease the blood viscosity, reduce myocardial oxygen consumption, and act as an antiarrhythmic. The combined use of these drugs could benefit the qi and promote the Yang, thus increasing the Yang and promoting blood circulation. Sufficient qi and blood could nourish the cardiac vessels, stop the palpitations, regulate the pulse, benefit the qi, nourish the blood, and promote the Yang.

Our previous studies showed that, in rabbit SSS models induced by ischemia-reperfusion, mRNA expression and HCN4 protein levels in the damaged SNC decreased significantly, suggesting that the development of SSS is closely associated with HCN4 protein expression.

Immunohistochemical examinations and reverse transcription polymerase chain reaction also showed that Yiqi Tongyang could significantly increase the HCN4 mRNA level in sinoatrial node tissues and that the distribution of HCN4 mRNA was homogeneous, suggesting that Yiqi Tongyang could increase the pacing function of the sinoatrial node by improving the HCN4 mRNA and protein levels within the damaged sinoatrial node [13].

The findings of the present study showed that the phosphorylated HCN4 protein expression in the* in vitro* ischemia-reperfusion model in SNC decreased significantly, as did the PKA activities in the SNC. However, the application of Yiqi Tongyang significantly increased the OD values of HCN4 protein phosphorylation as well as cellular PKA activities. The findings of* in vivo* and* in vitro* experiments showed that Yiqi Tongyang could increase *I*_*f*_ by upregulating the expression of phosphorylated HCN4 channel protein in the SNC, which improves SNC automaticity and thus improves the heart rate and ameliorates the SSS.

## 5. Conclusions

*I*_*f*_ is an important current in cardiac pacing. The abnormal phosphorylation of *I*_*f*_ channel protein HCN4 is the molecular basis for the development of SSS; and the PKA signaling pathway affects the development of SSS.

## Figures and Tables

**Table 1:** Optical density values of the HCN4 protein expression and phosphorylation in the rabbit sinoatrial node cells in each group (mean ± standard division, *n* = 3).

Group	HCN4 (%)	P-HCN4 (%)
Control group	100.00^*∗*^	100.00^*∗*^
Model group	46.72 ± 5.34	42.47 ± 4.96
Serum control group	55.14 ± 3.96^△^	37.88 ± 6.24^△^
Low-dose group	55.91 ± 6.27^△^	45.46 ± 10.57^△^
Moderate-dose group	106.36 ± 9.81^*∗*^	114.55 ± 7.55^*∗*^
High-dose group	122.29 ± 13.86^*∗*^	129.14 ± 12.95^*∗*^

^*∗*^ *P* < 0.05 and ^△^ *P* > 0.05 compared with the model group.

**Table 2:** PKA activities in the rabbit SNC in each group (mean ± standard division; *μ*mol NADH/min).

Group	PKA activities
Control group	0.18 ± 0.04
Model group	0.03 ± 0.00^*∗*^
Serum control group	0.03 ± 0.00^*∗*^
Low-dose group	0.05 ± 0.00^*∗*△^
Moderate-dose group	0.06 ± 0.00^*∗*△^
High-dose group	0.11 ± 0.01^*∗*△^

PKA: protein kinase A; ^*∗*^ *P* < 0.05 compared with the control group; ^△^ *P* < 0.05 compared with the model group.
